# PKCε Stimulated Arginine Methylation of RIP140 for Its Nuclear-Cytoplasmic Export in Adipocyte Differentiation

**DOI:** 10.1371/journal.pone.0002658

**Published:** 2008-07-16

**Authors:** Pawan Gupta, Ping-Chih Ho, M. D. Mostaqul Huq, Amjad Ali Khan, Nien-Pei Tsai, Li-Na Wei

**Affiliations:** Department of Pharmacology, University of Minnesota Medical School, Minneapolis, Minnesota, United States of America; Texas Tech University Health Sciences Center, United States of America

## Abstract

**Background:**

Receptor interacting protein 140 (RIP140) is a versatile transcriptional co-repressor that plays roles in diverse metabolic processes including fat accumulation in adipocytes. Previously we identified three methylated arginine residues in RIP140, which rendered its export to the cytoplasm; but it was unclear what triggered RIP140 arginine methylation.

**Methodology/Principal Findings:**

In this study, we determined the activated PKCε as the specific trigger for RIP140 arginine methylation and its subsequent export. We identified two PKCε–phosphorylated residues of RIP140, Ser-102 and Ser-1003, which synergistically stimulated direct binding of RIP140 by 14-3-3 that recruited protein arginine methyl transferase 1 to methylate RIP140. The methylated RIP140 then preferentially recruited exportin 1 for nuclear export. As a result, the nuclear gene-repressive activity of RIP140 was reduced. In RIP140 null adipocyte cultures, the defect in fat accumulation was effectively rescued by the phosphoylation-deficient mutant RIP140 that resided predominantly in the nucleus, but less so by the phospho-mimetic RIP140 that was exported to the cytoplasm.

**Conclusions/Significance:**

This study uncovers a novel means, via a cascade of protein modifications, to inactivate, or suppress, the nuclear action of an important transcription coregulator RIP140, and delineates the first specific phosphorylation-arginine methylation cascade that could alter protein subcellular distribution and biological activity.

## Introduction

Receptor-interacting protein 140 (RIP140) also known as NRIP1 (Nuclear Receptor Interacting Protein 1) is a versatile co-repressor for nuclear receptors and many transcription factors and contains several autonomous repressive domains [1]–[5]. RIP140 is known to play important roles in adipocyte and hepatocyte function [6]–[7], energy homeostasis [8] and reproduction [9], as well as a wide spectrum of metabolic pathways such as glucose uptake, glycolysis, TCA cycle, fatty acid oxidation, mitochondrial biogenesis and oxidative phosphorylation, etc. [10]. In RIP140-null mice, a repertoire of genes were found to be abnormally expressed [6], [11]. Our recent endeavors further uncovered extensive post-translational modifications (PTMs) of RIP140, which not only altered the property and function of RIP140 but also triggered its specific subcellular translocation [5], [12].

Established PTMs of RIP140 include phosphorylation [4], [13], acetylation [14], methylation [12] and pyridoxal 5′-phosphate (PLP) conjugation [15]. All these PTMs affect the biological activity of RIP140. Of particular significance is protein arginine methyl transferase (PRMT)-mediated arginine methylation on three specific arginine residues of RIP140, Arg240, 650, 948, which negatively regulates its biological activity in the nucleus (gene repression) by reducing its interaction with a corepressive enzyme machinery containing HDAC3 and facilitating its export to the cytoplasm via the exportin (CRM1)-containing export machinery [12]. As a result, the nuclear, gene repressive, activity of RIP140 is reduced.

Some of the extracellular cues/stimuli for phosphorylation, acetylation and pyridoxylation of RIP140 have begun to be investigated, but it was unclear what triggered protein arginine methylation of RIP140 to stimulate its nuclear export. In particular, the signals for this important PTM in the context of adipocyte differentiation, where the physiological role of RIP140 has been clearly established, was most interesting. The primary goals of this study were to identify the signal(s) that stimulated arginine methylation of RIP140 in adipocyte differentiation, and to delineate the signal transduction pathway that transmitted the stimuli and ultimately rendered the export of RIP140 to the cytoplasm.

It was first found that protein kinase C epsilon (PKCε)-stimulated phosphorylation of RIP140 also reduced its gene repressive activity by triggering its export to the cytoplasm. The targets of PKC action on RIP140 were located to two specific serine residues, Ser-102 and 1003, both of which were critical for its arginine methylation and nuclear export. This was mediated by the recruitment of chaperone 14-3-3/PRMT1 complex to the PKC-phosphorylated RIP140 and the action of PRMT1 to methylate RIP140. In differentiating cultures, the expression and activity of PKCε was elevated, which triggered this cascade of events. Further, the physiological relevance of this signal transduction pathway, that altered the PTMs of RIP140 and its subcellular localization and activity, was demonstrated in gain- and loss-function studies using specific point mutations, as well as wild type and RIP140 null adipocyte cultures.

## Results

### PKC-stimulated phosphorylation at Ser-102 and Ser-1003 of RIP140 regulates its cytoplasmic localization

**Figure 1:**
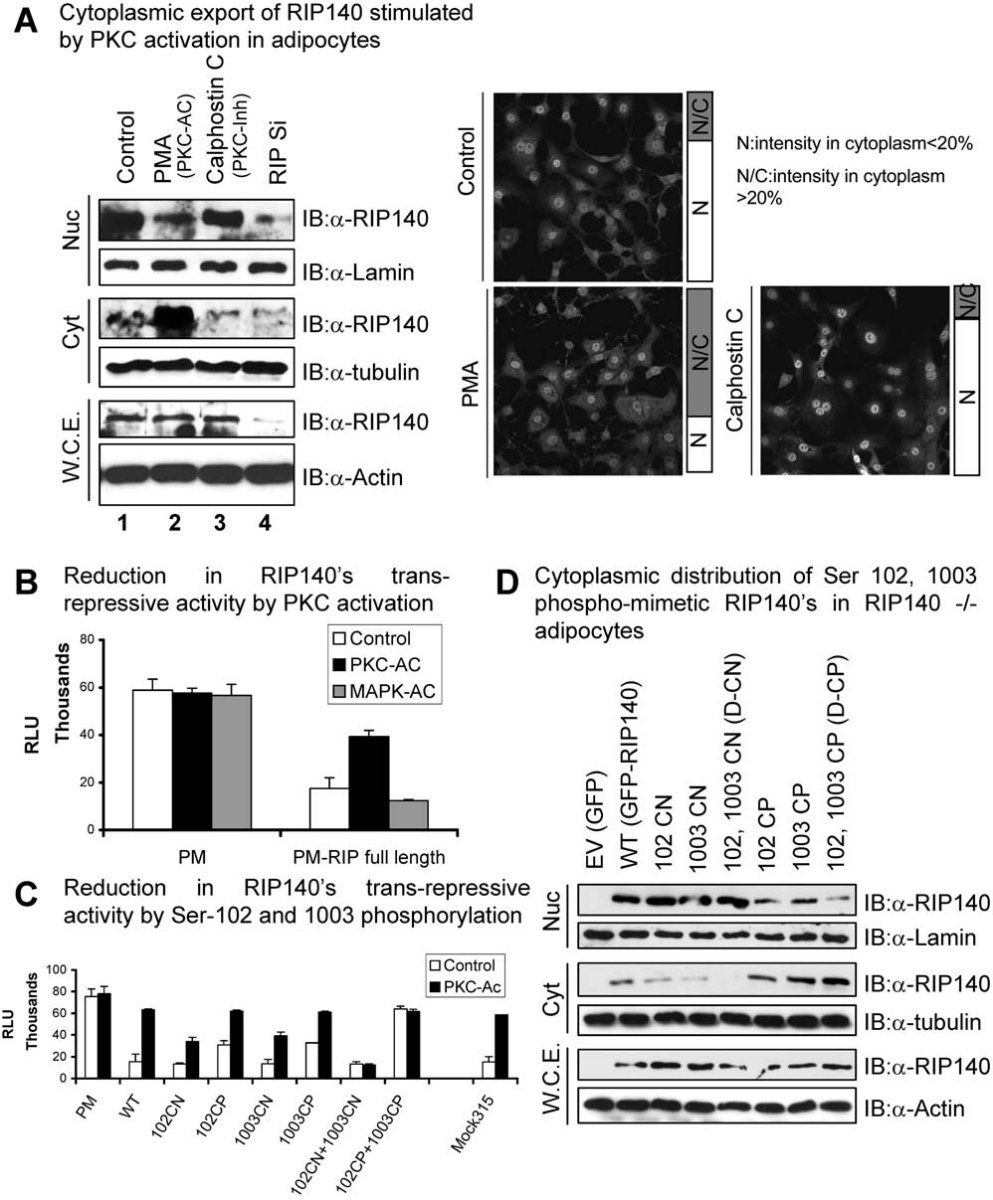
Effect of PKC phosphorylation on subcellular localization of RIP140. (A) Cellular distribution of endogenous RIP140 in differentiated adipocyte cultures where PKC activity was pharmacologically activated (PMA) or inactivated (Calphostin C). The results were obtained in cell fractionation studies (left) and immunocytochemistry (right). (B) PMA (PKC-Ac) reduced nuclear trans-repressive activity of RIP140. (C) Ser-102 and Ser-1003, residues mediated, synergistically, the effect of PKC on trans-repressive activity of RIP140. (D) Sub-cellular distribution of single/double constitutive negative (CN)/phospho-mimetic (CP) mutants. D-CN: double constitutive negative mutant; D-CP: double constitutive (phospho-mimetic) mutant.

In our previous study, [12] we have established the export of RIP140 to the cytoplasm, which was stimulated by its specific arginine methylation. To examine the upstream signaling events that triggered the export of RIP140, a preliminary study was conducted by using pharmacological agents. These tests revealed that compounds that regulated PKC activities could modulate the nucleo-cytoplasmic translocation of endogenous RIP140 in differentiating (8 days) adipocyte cultures (Figure 1A). A fraction of endogenous RIP140 appeared in the cytoplasm of the normally differentiating culture (lane 1, control), and a significantly increased cytoplasmic RIP140 was detected when the PKC pathway of the culture was further stimulated (lane 2). Conversely, RIP140 remained, primarily, nuclear when the PKC pathway was blocked (lane 3). An RNAi experiment to knock down endogenous RIP140 was included for a control (lane 4). In agreement with PKC-stimulated cytoplasmic localization of RIP140, the known biological activity of nuclear RIP140, gene repression, was inhibited by PKC but not MAPK (a negative control) (Figure 1B). Further it was the N- and C-termini, but not the central domain, of RIP140 that responded to the activation of PKC (Figure S1A).

To identify residues responsive to PKC activation, we extended our previous proteomic endeavor that has revealed eleven phosphorylated sites on RIP140 [4], [13] and uncovered one additional phosphorylated residue at Ser-102 (Figure S2). Since both the N- and the C-termini, but not the central domain, responded to PKC activation, we then screened all the phosphorylated residues in these two domains, including residues at 102, 104, 202, 207, 358, 380 (for the N-terminus) and a single phosphorylated residue at 1003 (for the C-terminus) by mutations mimicking constitutive dephosphorylation (CN, S/T→A). This series of mutation studies identified Ser-102 and Ser-1003 as the critical residues responsive to PKC activation (Figure S1B). The functional roles of these two residues, with respect to gene repression, were further verified by incorporating either the constitutive negative (dephosphorylation, CN, S→A) or the phospho-mimetic (CP, S→E) [4], [16] mutation in the context of the full-length protein. (Figure 1C). It appeared that while each single mutation of 102, or 1003, exerted a partial effect, only the double mutation (of Ser-102 and Ser-1003) could completely abrogate the responsiveness to PKC activation, indicating synergistic effects of phosphorylation at these two specific residues. The double negative mutant (102CN+1003CN) was strongly repressive, whereas the double phospho-mimetic mutant (102CP+1003CP) was not effective in trans-repression. A mock mutation at Ser-315, a negative control, exerted no effect on the repressive activity of RIP140.

We then validated the effects of phosphorylation of Ser-102 and Ser-1003 on subcellular distribution of RIP140 in reconstituted RIP140 null adipocyte cultures. While the individual mutation, either Ser-102 or Ser-1003, slightly affected the nuclear/cytoplasmic distribution of RIP140, only the double mutant exhibited a more pronounce effect (Figure 1D). The double negative (D-CN) mutant was retained, almost entirely, in the nucleus whereas the double phospho-mimetic mutant (D-CP) was increasingly cytoplasmic. This was in agreement with the result of their trans-repressive activity shown in Figure 1C.

### PKC-phosphorylated RIP140 is associated with 14-3-3 and PRMT1 and is methylated at arginine residues

**Figure 2:**
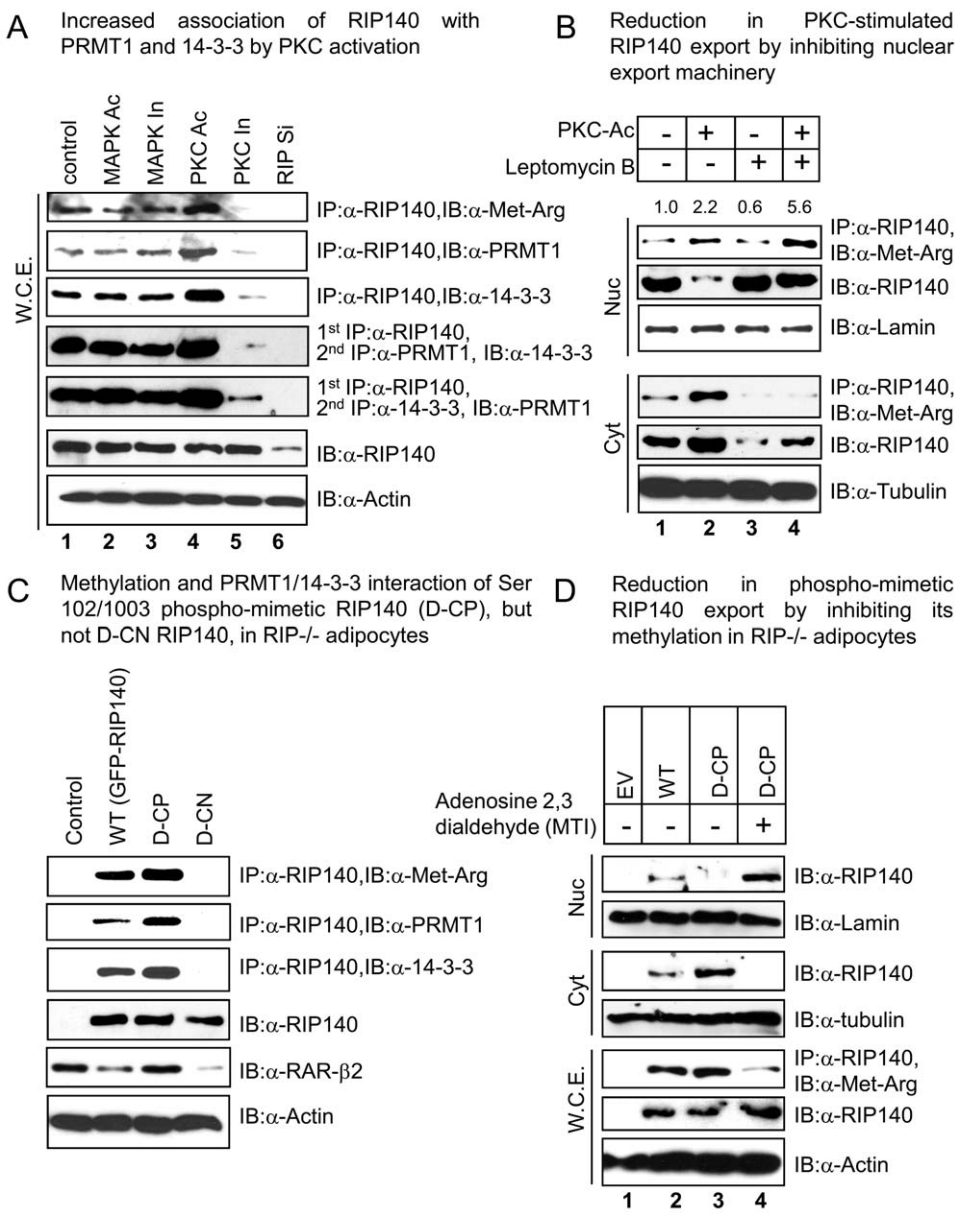
Effects of PKC-mediated phosphorylation on RIP140 interaction with the export effectors. (A) PKC, but not MAPK, activation induced *in vivo* hypermethylation and association of endogenous RIP140 with the effecter molecules 14-3-3 and PRMT1. (B) Leptomycin B (LMB), a specific inhibitor of CRM1, blocked PKC triggered export of RIP140 without affecting its methylation status. (C) D-CP, but not D-CN, RIP140 was methylated and associated with the effecter molecules 14-3-3 and PRMT in RIP140 null adipocytes. The nuclear activity of the WT, D-CN, D-CP RIP140 was verified by examining the repression of its endogenous target RAR-β2 detected on a western blot. (D) A methylation inhibitor (MTI) blocked the export of D-CP RIP140.

In our previous study we identified three methylated arginine residues on RIP140, which stimulated its export to the cytoplasm [12]. A separate study from another group has implicated 14-3-3 dependent intracellular relocalization of RIP140 in the cytoplasm [17]. It was known that 14-3-3 and PRMT1 could directly interact with multiple common partners such as DAL-1 [18]–[19], p53 [20]–[21], and histone 4 [22]–[23]; therefore, 14-3-3 and PRMT1 could possibly exist in the same molecular complex. We thus speculated if PKC-stimulated phosphorylation of RIP140 could trigger its physical association with the 14-3-3/PRMT1 complex, thereby rendering its arginine methylation (Figure 2A). Very interestingly, in differentiated adipocyte cultures, a basal methylation level of endogenous RIP140 was detected in the absence of any external stimuli, supporting the presence of an autonomous signaling pathway in the differentiating adipocyte culture for arginine methylation of RIP140 (Figure 2A top panel, lane1). While additional MAPK stimulation (lane 2) did not significantly affect the methylation status of RIP140, additional PKC activation was able to enhance (lane 4), and inhibition of PKC reduced (lane 5), the level of arginine methylation of endogenous RIP140. Consistently, complex formation of RIP140/PRMT1, and of RIP140/14-3-3, was significantly enhanced by a PKC activator and reduced by a PKC inhibitor (2^nd^ and 3^rd^ panels). The formation of the tri-molecular complex was then verified by sequential IP experiments where the RIP140/PRMT1 or RIP140/14-3-3 immuno-complex was subsequently examined to determine its association with the third partner, 14-3-3 or PRMT1 (4^th^ and 5^th^ panels). These results, all together, demonstrated PKC activation-stimulated tri-molecular complex formation of endogenous RIP140/14-4-3/PRMT1 in differentiating adipocyte culture, which rendered arginine methylation of RIP140.

It was known that the export of RIP140, as stimulated by PRMT1 mediated methylation, involved a down stream effecter, the exportin (CRM1) [12]. In order to determine if PKC-triggered export of RIP140 could also be attributed to the action of exportin CRM1, a selective inhibitor of CRM1-mediated export, leptomycin B, [24] was used. As shown in Figure 2B lane 2, arginine methylation of RIP140, as well as its cytoplasmic distribution, was increased by the activation of PKC. Leptomycin B significantly hindered PKC-stimulated export of RIP140, with methylated RIP140 accumulated in the nucleus (lane 4), suggesting that PKC-activated export of RIP140 employed the same CRM1-mediated export machinery.

The PKC-triggered phosphorylation-methylation cascade and its associated effector molecules were then validated in RIP140 null adipocytes rescued by expressing either the dominant negative (D-CN) or the double phospho-mimetic (D-CP) mutant RIP140. The wild type RIP140 was methylated, and was able to interact with PRMT1 and 14-3-3 at a basal level. For the phospho-mimetic RIP140, methylation was enhanced and its association with 14-3-3 and PRMT1 was also increased. On the contrary, the dominant negative mutant failed to be methylated and could not be associated with PRMT1 or 14-3-3 (Figure 2C). In agreement with the methylation status of RIP140, which is a key actor regulating cellular distribution of RIP140 [12], the gene-repressive (nuclear activity) effect of the wild type and the mutant RIP140 on one of the endogenous target genes, RAR-β2, was verified as shown in Figure 2C (the 2^nd^ panel from the bottom). The wild type (WT), and the dominant negative (D-CN) RIP140 which was predominantly nuclear in localization, repressed basal expression of RAR-β2; whereas the double phospho-mimetic (D-CP) RIP140, which would be primarily cytoplasmic, did not elicit such a nuclear activity. We further verified if methylation on the phospho-mimetic RIP140 was important for its export by using adenosine-2,3-dialdehyde, a global methyl transferase inhibitor (MTI), in this system. This methylation inhibitor abrogated the methylation and export of the phospho-mimetic RIP140 (Figure 2D, lane 4), supporting arginine methylation as the down stream mediator of PKC phosphorylation-stimulated export of RIP140.

### 14-3-3 chaperone facilitates the recruitment of PRMT1 to RIP140 for its methylation and export

**Figure 3:**
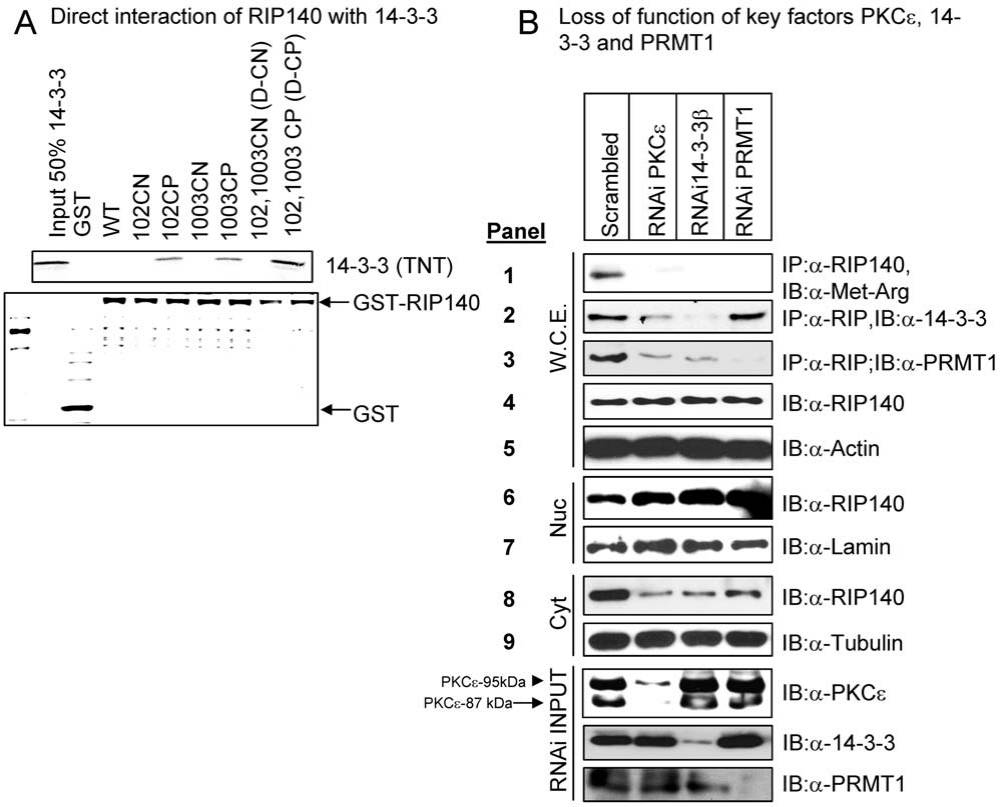
Functional roles of PKCε, 14-3-3 and PRMT1 in RIP140 export. (A) Direct interaction (GST pull down assay) of various GST-RIP140 wild-type/mutants with 14-3-3. D-CP mutant RIP140 (phospho-mimetic mutation on Ser-102 and Ser-1003) interacted strongly with 14-3-3. (B) Loss of function (RNA interference) of key molecules PKCε, 14-3-3β and PRMT1 in differentiating adipocyte cultures. A hierarchy of events involving PKCε phosphorylation of RIP140, direct binding of 14-3-3, and recruitment of PRMT1 to RIP140 for its subsequent methylation and export.

Ser-102 and 1003 were the principal targets of PKC that modulated the association of RIP140 with 14-3-3/PRMT1 (Figure 2). Ser-1003 is in close proximity to a consensus site for 14-3-3 binding, and Ser-102 is within an imperfect site for 14-3-3 binding (Table 1). Because RIP140 did not directly interact with PRMT1 [12], we thus speculated a role for 14-3-3 in mediating the formation of RIP140/14-3-3/PRMT1 tri-molecular complex. A GST-pull down assay was first employed to examine the potential direct interaction of 14-3-3 with RIP140 (Figure 3A). The bacterially produced wild type (WT) RIP140, as well as negative phospho-mutants (102CN and 1003CN single mutants and the double mutant D-CN), failed to interact with 14-3-3. Importantly, two single phospho-mimetic mutants (102CP and 1003CP) apparently interacted directly with 14-3-3; further, the double phospho-mimetic RIP140 (D-CP) even more strongly interacted with 14-3-3. This result clearly showed that while the dephosphorylated (at two PKC sites, residues 102 and 1003) RIP140 could not directly interact with 14-3-3, phosphorylation at these two sites indeed facilitated direct interaction of RIP140 with 14-3-3.

**Table 1:** PKC responsive sites of RIP140 and their consensus motif-specific chaperones.

Residue	Sequence^a^	Kinase/chaperone^b^
Ser-102	KRKRL**S**DSIVN	PKC, 14-3-3 ?
Ser-1003	DHRTF**S**YPGMV	PKC, 14-3-3

Twelve phosphorylation sites of RIP140 purified from insect cell cultures have been identified by LC-ESI-MS/MS analysis. Ser-102 in N-terminal and Ser-1003 in C-terminal are consensus and responsive to PKC and 14-3-3 binding.

a Sequences adjoining to the residues were compared to consensus motifs of known kinases/chaperone.

b PKC: S/T-X-K/R, K/R-X-X-S/T, K/R-X-S/T; 14-3-3: R(S)X_1,2_pSX(P), RXX(pS/pT)XP and RXFX(pS/pT)XP where S, T, K, R, and P are single-letter codes of amino acids, and X can be any amino acid. Small case p refers to phosphorylation at respective residue.

The results thus far have suggested a signaling pathway, initiated from the activated PKC that triggered RIP140 phosphorylation at residues 102 and 1003, stimulated RIP140's direct interaction with (or recruitment of) 14-3-3, which could scaffold PRMT1. The recruited PRMT1 then acted on RIP140 to methylate three arginine residues [12], resulting in the export of methylated RIP140. It has been reported that in differentiated adipocytes, PKCε was the principal functional PKC [25]–[27], which had also been detected in our experimental system. A knockdown approach was then employed to validate the speculated functional roles of the key factors in this pathway, PKCε, 14-3-3 and PRMT1 (Figure 3B). The results showed that knockdown of any one of these three factors indeed drastically reduced methylation (the 1st panel) and export (the 8^th^ panel) of RIP140, confirming the functional roles for these factors in this signal transduction pathway. Further, knockdown of PKCε abrogated the association of RIP140 with 14-3-3 (the 2^nd^ panel) and PRMT1 (the 3^rd^ panel). Very interestingly, while knockdown of 14-3-3 reduced the association of RIP140 with PRMT1 (the 3^rd^ panel), knockdown of PRMT1 had no effect on the association of RIP140 with 14-3-3 (the 2^nd^ panel). These results supported a hierarchy of events initiated from PKCε-mediated phosphorylation, which triggered the recruitment of 14-3-3 that could scaffold PRMT1 to methylate RIP140, thereby stimulating its subsequent nuclear export.

### Kinetics of changing factors stimulating arginine methylation of RIP140 in differentiating adipocytes

**Figure 4:**
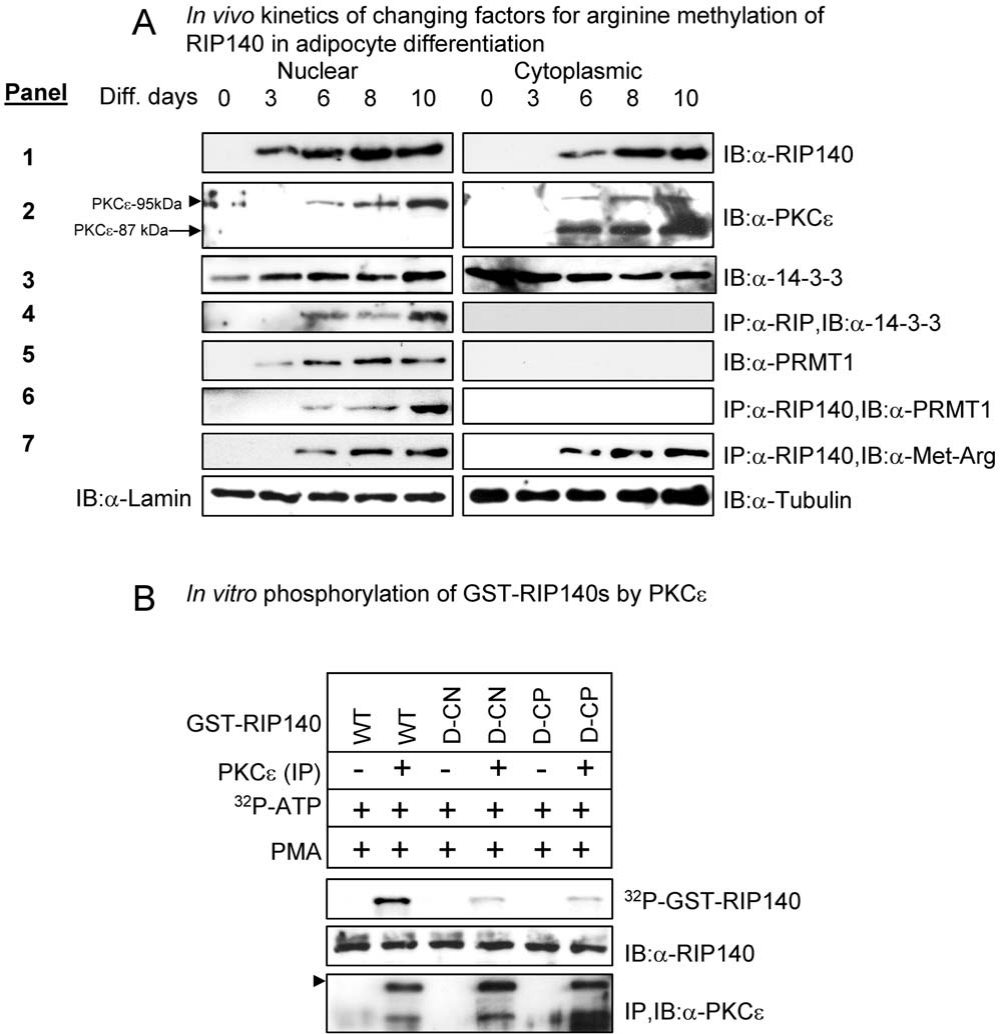
Kinetics of cellular factors stimulating RIP140 export. (A) Kinetics of the expression/distribution of the regulatory molecules PKCε, 14-3-3β and PRMT1, leading to RIP140 export in the adipocyte differentiation model. (B) *In vitro* phosphorylation of RIP140 (purified from bacteria) by endogenous PKCε partially purified from differentiating adipocyte cultures.

To examine the changing cellular factors leading to ultimate arginine methylation of RIP140 in differentiating adipocyte culture, the kinetics of the expression and/or activation of relevant endogenous factors, as well as the nuclear/cytoplasmic distribution of these components and RIP140 in differentiating adipocyte culture were examined as shown in Figure 4A. As expected, RIP140 was not detected in the undifferentiated pre-adipocyte culture, readily appeared in the nucleus at the first time point examined (day 3) and then was detected in the cytoplasm at the second time point examined (day 6) of the differentiating culture (top panel). The expression of PKCε was clearly detected at day 6 of differentiation and steadily increased (2^nd^ panel). PKCε migrated on SDS/PAGE predominantly as a doublet with molecular masses of 87 kDa and 95 kDa (PKCε^87^ and PKCε^95^, respectively) [28]. While the nuclear fraction associated PKCε corresponded to PKCε^95^ (2^nd^, left panel), the cytoplasmic PKCε was, primarily, PKCε^87^ (2^nd^, right panel). We have also verified the activity of nuclear PKCε *in vitro* (see the following, Figure 4B and Figure S3). Regarding 14-3-3β, others have shown its expression in adipocytes [29]. Interestingly while 14-3-3β was detected in both the nucleus and the cytoplasm of the differentiating culture (3^rd^ panel), it was associated with RIP140 only in the nucleus (4^th^ panel). PRMT1 was restricted to the nucleus as also shown in a previous report [30] (5^th^ panel) and was associated with RIP140 in the nucleus (6^th^ panel). The overall kinetics of the association of RIP140 with 14-3-3 (4^th^ panel) and PRMT1 (6^th^ panel) agreed with that of PKCε activation in this experimental system, and was in line with the kinetics of arginine methylation of RIP140 (7^th^ panel), and ultimately its export (top panel).

PKCε was found to be the principal functional isoform of PKC expressed in the later stages during adipocyte differentiation [25]–[27]. To verify the enzyme activity of PKCε on RIP140, an *in vitro* phosphorylation assay was conducted (Figure 4B). While the wild type RIP140 expressed in bacteria could be phosphorylated by the partially purified endogenous PKCε collected from differentiating adipocytes (lane 2), the dominant negative (CN) RIP140 and the PKC phospho-mimetic (CP) RIP140 were only marginally phosphorylated, supporting Ser-102 and 1003 as the principal targets of PKCε mediated phosphorylation. This was consistent with the response of these mutants to PKC activation shown previously (Figure 1B & C, Figure S1). To verify the activity of PKCε in the nuclear and cytoplasmic fractions, an *in vitro* phosphorylation assay was conducted using partially purified PKCε isolated from either the nuclear or the cytoplasmic fraction of differentiating adipocyte culture that was further activated *in vitro* by PMA (Figure S3). While PKCε from the nuclear fraction was able to readily phosphorylate RIP140 even without *in vitro* activation by PMA, the principal cytoplasmic PKCε (PKCε^87^) required *in vitro* activation by PMA to act on RIP140, supporting the notion that PKCε^95^ in the nuclear fraction, but not the cytoplasmic PKCε^87^, was catalytically active in these cells [28]. This could be attributed to the available cofactors DAG and phosphatidylserine in the nuclear/perinuclear region [28], [31]–[32] but not in the cytoplasm. This result confirmed the phosphorylation of RIP140 by the functional PKCε in the nuclear or perinuclear regions, leading to the subsequent signaling cascade that ultimately triggered the export of RIP140.

### Physiological relevance of PKCε activated RIP140 phosphorylation and its export with regards to fat accumulation in differentiated adipocytes

**Figure 5:**
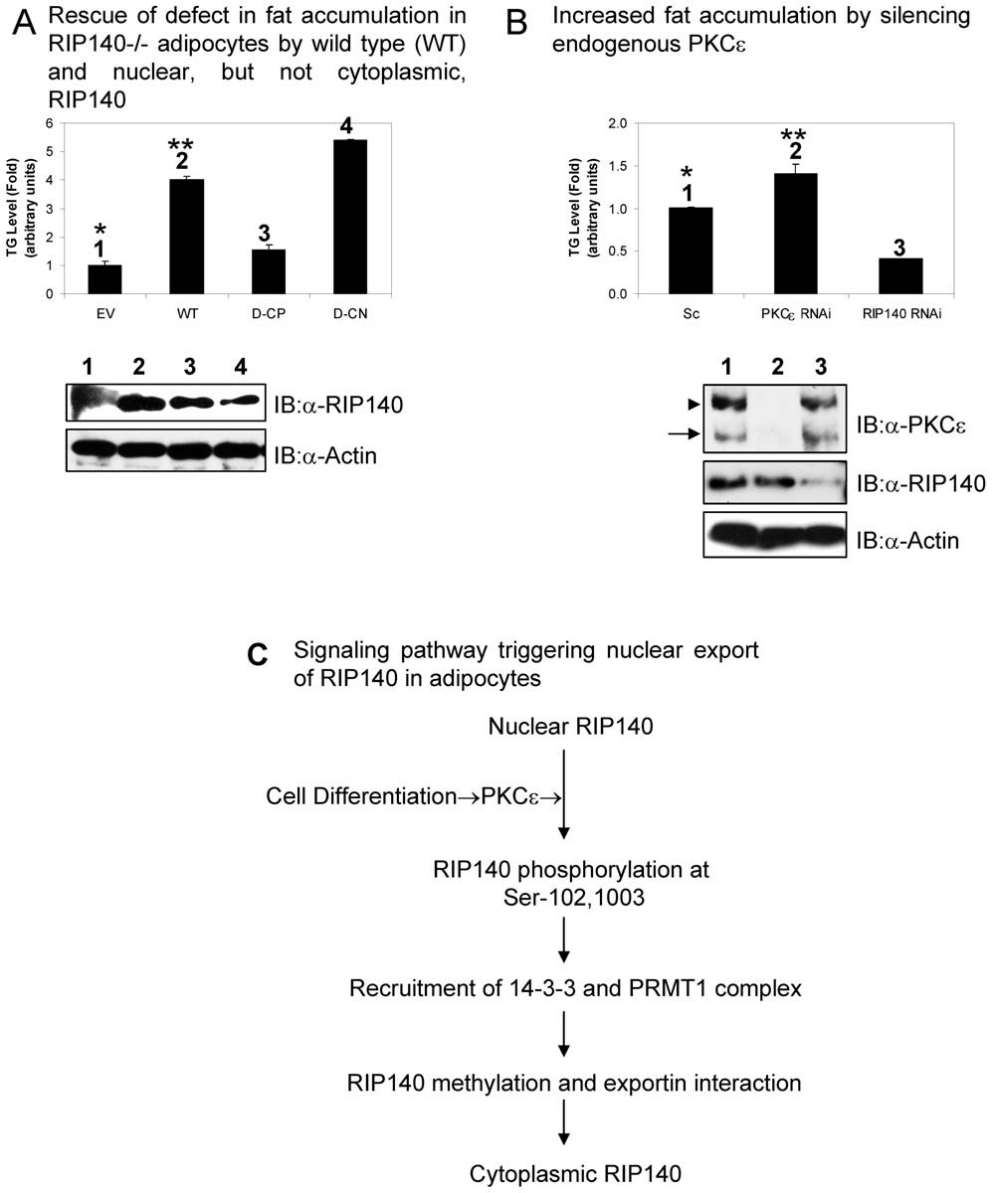
Physiological relevance of the sub-cellular distribution of RIP140 with respect to fat accumulation in adipocytes. (A) Gain of the nuclear (D-CN), but not the cytoplasmic RIP140 (D-CP), more effectively rescued the defect of fat accumulation in RIP140 null adipocyte cultures. **versus* p<0.05. (B) Loss of function of PKCε (to enhance nuclear RIP140) leads to higher levels of fat accumulation in differentiated adipocytes. **versus* p<0.05. (C) A schematic diagram depicting the signal transduction pathway of PKCε activation to the export of RIP140.

RIP140 was shown to be important for fat accumulation in adipocytes [6], [33], and its biological activity was attributed, primarily, to its effect on gene regulation, a nuclear event. The signals leading to its cytoplasmic export would likely reduce, or terminate, this nuclear effect, and possibly negatively regulated fat accumulation in differentiating or differentiated adipocytes. To test this hypothesis in the RIP140 null background, gain-of-function experiments were carried out as shown in Figure 5A. Re- expressing a wild type RIP140 (WT) effectively rescued the defect of fat accumulation in the knockout culture (Figure 5A, bar 2), but re-expressing the phospho-mimetic RIP140 (D-CP which could be exported) failed to efficiently rescue this defect (Figure 5B, bar 3). As predicted, re-expressing the phosphorylation mutant (D-CN which would be mostly nuclear) (Figure 5B, bar 4) rescued the defect as efficiently as the wild type RIP140. We also conducted loss- and gain-of-function studies of PKCε in the differentiating adipocyte culture (Figure 5B). As compared to the level of relatively efficient fat accumulation in the wild type culture, a further increase in fat accumulation was indeed detected in the culture with its endogenous PKCε silenced (Figure 5A, bar 2). Consistent with the result of studies of RIP140 gene knockout, knockdown of RIP140 also led to a significantly reduced level of fat accumulation (Figure 5A, bar 3). This result supported a physiological relevance of PKCε-triggered phosphorylation in the nuclear-cytoplasmic export of RIP140, which negatively regulated fat accumulation in differentiating or differentiated adipocytes.

## Discussion

This study extends our previously reported finding of arginine methylation on RIP140 that triggers its export to the cytoplasm in differentiating adipocytes, and provides mechanistic insights into the signal that triggers the activation of a specific pathway that ultimately leads to the action of PRMT1 to methylate RIP140. We establish the activated PKCε in differentiating adipocytes as the initial nuclear trigger that stimulates phosphorylation on two specific residues of nuclear RIP140 (on Ser-102 and Ser-1003), which renders the recruitment of a specific chaperon 14-3-3 to RIP140 in the nucleus that brings the associated PRMT1 to RIP140, thereby methylating RIP140 and stimulating its export. The functionality of the three major players (PKCε, 14-3-3 and PRMT1) in this signal transduction pathway has been confirmed in the differentiating adipocyte culture. This study reveals not only an autonomously activated signaling pathway for post-translational modifications to regulate the nuclear activity of an important coregulator for gene expression, RIP140, but also implicates a possibility that RIP140 may perform certain cytoplasmic functions in differentiating or differentiated adipocytes. With regards to the relationship of the kinetics of the signaling factors and RIP140 phosphorylation-methylation as shown in Figure 4A, we have observed interesting kinetics of these factors that stimulate phosphorylation and methylation of RIP140 in differentiating preadipocyte cultures. The changes of these factors are likely a result of cell-autonomous response in the early differentiation process rather than the outcome of phosphorylation-methylation of RIP140, because these factors are the upstream signals triggering phosphorylation-methylation of RIP140. It is therefore predicted that these factors will probably not drastically be affected by null mutation of RIP140, or mutation at phosphorylation-methylation of RIP140. On the contrary, the biological end point, or the effect of phosphorylation-methylation triggered nuclear export or RIP140, i.e. fat accumulation, indeed could be significantly affected by mutation in the modification status of RIP140 as shown in Figure 5A.

It is very interesting that RIP140 can be phosphorylated on at least 12 sites, but only Ser-102 and Ser-1003 are critical for the subsequent recruitment, or binding, of RIP140 to 14-3-3. This is in agreement with previous reports showing that the N-terminal and C-terminal domains of RIP140 are important for 14-3-3 binding [17]. 14-3-3 mediated cytoplasmic localization has been implicated, mostly, in the cytoplasmic retention of certain proteins [34]–[35], although several reports suggested its role also in nuclear export [36]–[38]. This could be due to the predominantly cytoplasmic distribution of 14-3-3 detected in certain cell types. In the adipocyte differentiation culture, 14-3-3 could be detected in both the nucleus and the cytoplasm, but its association with RIP140 occurred only in the nucleus (Fig. 4A). Further, 14-3-3 binding per se, i.e. without methylation (Figure 2D), failed to stimulate cytoplasmic distribution of RIP140, suggesting an active role for the methylation-dependent nuclear export of RIP140, rather than 14-3-3 mediated cytoplasmic retention or sequestration, of RIP140. It is likely that 14-3-3 serves as a chaperone to recruit PRMT1 to the phosphorylated RIP140 in the nucleus. PRMT1 methylates RIP140 and the methylated RIP140 then recruits exportin to mediate its nuclear export. This is in agreement with the widely recognized role for 14-3-3 as a scaffold [17], [39].

There is an absolute requirement for CRM1 to trigger RIP140 export, since the process is blocked by a selective inhibitor of CRM1, leptomycin B (Figure 2B). Arginine methylation of RIP140 is crucial since a PKC phospho-mimetic RIP140 (D-CP) failed to be exported in the presence of a methylation inhibitor (MTI) (Figure 2D). Binding by 14-3-3 is also indispensable for the export of RIP140 (Figure 3B). Thus, the control for cytoplasmic distribution of RIP140 involves a specific cascade of events to ensure proper transport of RIP140 between the nucleus and the cytoplasm. The employment of such a tightly controlled pathway to regulate RIP140 translocation within a specific time window of adipocyte differentiation strongly suggests certain important cytoplasmic functions or roles for RIP140 as the differentiating adipocyte cultures gradually change their properties.

RIP140 is a ligand dependent co-repressor of genes regulated by hormones and is shown to play important roles in adipocyte differentiation, in particular with regards to fat accumulation [6], [10]. Consistently, the cytoplasmic form of RIP140 (the CP mutant) could barely rescue the defect in fat accumulation whereas the nuclear form (the CN mutant) was fully capable of doing so. It is quite possible that the cytoplasmic form of RIP140 could play roles in events other than the fine-tuning of fat accumulation in differentiated adipocytes. RIP140 modulates diverse pathways such as glucose uptake, glycolysis, TCA cycle, fatty acid oxidation, mitochondrial biogenesis and oxidative phosphorylation, etc [7], [33]. It remains to be seen which one(s) of this spectrum of biological processes involves the cytoplasmic functions of RIP140.

Finally, phosphorylation has been reported to modulate modifications like acetylation [40], SUMOylation [41] and ubiquitination [42]. This current report is the first demonstrating a specific phosphorylation-methylation cascade that has an important biological consequence in differentiating adipocytes.

## Materials and Methods

### Plasmid constructs and site directed mutagenesis

Mouse complementary DNAs for GFP/GST/Gal4BD fused RIP140 and GAL4-tk-luciferase reporter were as described previously [2], [4]. Constitutive negative/positive, point/sequential mutations involving residues Ser-102, Ser-104, Thr-202, Thr-207, Ser-315, Ser-358, Ser380 and Ser-1003 in RIP140 expression vectors as template were made using QuikChange XL site-directed mutagenesis kit (Stratagene). The mutagenic primers for RIP140 were designed to match alanine (A) or glutamic acid (E). S/T→A produced a constitutively dephosphorylated state (CN), S/T→E mimicks a constitutively phosphorylated state (CP) [16]. The mutagenic primers are: S102A: 5′-CGGAAGAGGCTGGCTGATGCCATCGTG-3′ (sense), 5′-CACGATGGCATCAGCCAGCCTCTTCCG–3′ (antisense); S1003A: 5′-CATAGGACATTTGCATACCCGGGAATG-3′ (sense), 5′-CATTCCCGGGTATGCAAATGTCCTATG-3′ (antisense); S102E: 5′-CGGAAGAGGCTGGAAGATTCCATCGTG-3′ (sense), 5′-CACGATGGAATCTTCCAGCCTCTTCCG-3′ (antisense); S1003E: 5′-CATAGGACATTTGAATACCCGGGAATGGT-3′ (sense), 5′-ACCATTCCCGGGTATTCAAATGTCCTATG-3′ (antisense); Mutagenic primers for other residues have been reported previously. The positive clones were verified by DNA sequencing and for expression.

### Cell culture, transfection and RNA interference

3T3-L1 and RIP140 null adipocytes were maintained and differentiated (8 day) by a differentiation cocktail that included insulin, triiodothyronine, dexamethasone and isobutylmethylxanthine as described earlier [43]. Transient transfection/ reconstitution in RIP null adipocytes was as described earlier [12]. Quantitative determination of triglycerides (TG) was performed by alkaline hydrolysis and measurement of glycerol released by the Free Glycerol Determination Kit (Sigma). The TG level was normalized to protein concentration. Nuclear and cytoplasmic fractions were obtained as described previously [44] with some modifications. Scrambled RNA and siRNAs for *Nrip1* (encoding RIP140; 5′-GCUUCUUUCUUUAAUCUAATT-3′/5′-UUAGAUUAAAGAAAGAAGCTT-3′, SI02698759), *Prkce* (encoding PKCε; 5′-GGCGGAACUCAAAGGCAAATT-3′/5′-UUUGCCUUUGAGUUCCGCCAA-3′, SI01388793) and *Ywhab* (encoding 14-3-3β; 5′-CCCUGAAUGAAGAGUCUUATT-3′/5′-UAAGACUCUUCAUUCAGGGTG-3′, SI00212037), were from Qiagen. siRNAs for *Prmt1* (encoding PRMT1) were from Dharmacon [12]. RNAs were introduced using DharmaFECT1 (T-2001-01, Dharmacon) or HiPerfect (no. 301704, Qiagen). Silencing was assessed by western blots at 72 hs.

### Chemicals and treatments

All treatments were done in Dulbecco's modified Eagle's medium containing DCC serum. Leptomycin B (18 nM), a selective inhibitor of CRM1, and adenosine 2,3 dialdehyde, a global methyl transferase inhibitor (10 µM) (Sigma), were added for 4 h before harvesting the cells. Activators/inhibitors (calbiochem) of kinases, Sphingosine-1-phosphate (MAPK/ERK activator, 1 µM), Phorbol-12-myristate-13-acetate (PKC activator, 10 nM), PD98059 (MAPK inhibitor, 3 µM), Calphostin C (PKC inhibitor, 50 nM) were added for 4 h unless otherwise stated.

### Immunoprecipitation and western blotting

Cell extracts were suspended in 250 µl of immunoprecipitation buffer (150 mM NaCl,50 mM Tris-HCl (pH 8.0), 1 mM EDTA, 0.2% (v/v) Nonidet P40, 2 mM PMSF, 0.1% (w/v) SDS and a protease-inhibitor cocktail. Protein extracts (200 µg) were incubated with antibodies to RIP140, 14-3-3, or PRMT1 overnight at 4°C, and precipitated with protein G–agarose beads for 1–2 hs. Western blots were performed as described [45]. Antibodies used were, RIP140 [46], PKCε (sc-214, Santa Cruz), 14-3-3β (sc-629, Santa Cruz), PRMT1 (sc-13393, Santa Cruz), RAR-β2 [12] and methylated-arginine specific antibody (ab412, Abcam). Reduced protein inputs were used for ectopic expression of RIP140 to avoid saturation.

### Immunohistochemical staining

For immunohistochemical staining, 3T3-L1 cells were differentiated for 8 days by differentiation protocol. Cells were treated as indicated in serum-free media. After 2 h, cells were fixed by a 4% fixation solution and permeated by a permeation buffer for 10 and 5 minute on ice, respectively. After permeation, cells were blocked by a blocking buffer (1% BSA and 0.1% Tween-20 in PBS) for 30 minute and then incubated with the primary antibody overnight in 4°C. After washing three times, cells were incubated with the secondary antibody for 2 h in room temperature. Images were acquired using Olympus fluoview 1000. The typical field containing multiple cells of the unprocessed images were cropped with a similar dimension for each image using the crop tool at Adobe Photoshop. To determine the differential sub-cellular distribution pattern of RIP140 constructs, many microscopic fields of the images were randomly selected with the help of a manipulator and the distribution pattern (nuclear, nuclear-cytoplasm, and cytoplasmic) within cells were recorded. In addition, the randomly selected fields were properly focused to capture the images with the same setting and stored as digital image files. The unprocessed images were retrieved for unbiased cell counting. Cytoplasmic intensity >20% of total intensity was counted as N/C.

### Analysis of Biological (Trans-repressive) Activity of RIP140

The technique for culturing COS-1 cells, transfection experiments, and luciferase and *lacZ* assay were as described previously [12]. Cells were transiently transfected with a mixture of pBD-GAL4-RIP140 full length/N-terminal, wild-type (WT)/mutant (Mut) (0.1 µg) or pBD-GAL4 (0.1 µg), GAL4- tk-luciferase (0.5 µg) reporter, and a CMV-*lacZ* internal control (0.05 µg). 20 h post-transfection, cultures were fed with fresh medium containing dextran charcoal (DCC)-treated serum and treated for 4 h with either MAPK or PKC activator. 24 h post-transfection, total cell extracts were collected and tested for luciferase and *lacZ* activity. The fold relative luciferase activity was calculated by normalizing each relative luciferase units (RLU) activity to the RLU of pBDGAL4. Reported values were the averages of three experiments with triplicate measurements.

### GST pull-down assays

Various GST–RIP140 wt/mt constructs [full-length (amino acids 1–1161), were expressed and purified from E. coli and bound to GST beads as described earlier [45]. Washed beads were incubated with *in vitro*-transcribed and translated (Promega) [^35^S] Met-labeled 14-3-3. Specifically bound proteins were resolved by SDS-PAGE and detected with a Phosphorimager. *In vitro* transcription-coupled translation product subjected to SDS-PAGE constituted TNT input (50%). Equal volumes subjected to SDS-PAGE and stained with Coomassie blue constituted the GST input.

### In vitro phosphorylation assay

PKCε was partially purified from adipocyte whole cell extracts and nuclear and cytoplasmic fractions by immunoprecipitation. *In vitro* phosphoylation of bacterial purified wt/mt RIP140 by PKCε was carried out as described [47].

### Purification and Mass Spectrometric Analysis of RIP140

To identify the phosphorylation sites on RIP140, we expressed the His-tagged RIP140 in insect cells as a eukaryotic host for mammalian protein expression. The protein was purified by affinity column over Talon resin with 95% homogeneity. The details of the procedure for RIP140 purification was described previously [13]. The purified RIP140 was subjected to tryptic digestion, and LC-ESI-MS/MS analysis was conducted as described [13].

## Supporting Information

Figure S1 Screening of PKC responsive domains and residues of RIP140 with regards to its trans-repressive activity. (A) PKC activation reduced transrepression mediated by the N- and C-terminal, but not the central, domain of RIP140. (B) Ser-102 (in the N-terminal domain) and Ser-1003 (in the C-terminal domain) were responsive to PKC modulation. PKC activator: PMA.
(0.23 MB TIF)

Figure S2 Mapping of phosphorylation sites on Ser-102 and Ser-104 on mouse RIP140 purified from insect cultures, by LC-ESI-MS/MS analysis. The total ion chromatogram (LC-MS) of tryptic digests of RIP140 showed three modified peptides spanning amino acids 101–111aa (101LSDSIVNLNVK111) (A), 101–112 aa (101LSDSIVNLNVKK112) (B), and 100–111 aa (100RLSDSIVNLNVK111) (C) contained both Ser-102 and Ser-104 (bold letter). The modified peptide spanning 100–111 (C, top) and 101–112 (B, top) appeared as doubly charged ions, respectively at 719.36 m/z (mol. mass 1436.72) and 705.35 m/z (mol. mass 140.72), while the peptide (101–111 aa) appeared as a triply charged ion at 641.31 m/z (mol. mass 1280.63). The precursor mass of each ion from the modified peptide showed +80 Da mass shift as compared to the each doubly charged peptide ion of the corresponding unmodified peptide 100–111 aa (679.37 m/z, mol. mass 1356.76 Da) (C, bottom), 101–111 aa (601.33 m/z, mol. mass 1200.66 Da) (A, bottom) and 101–112 aa (665.36 m/z, mol. mass 1328.75 Da) (B, bottom). This indicated that each peptide is modified by a mono-phosphorylation site. Previously, by MS/MS analysis of the precursor ion of the modified peptide 100–111 aa (C, top), we have reported the assignments of phosphorylation site at Ser-104 (Huq et al, 2005). However, careful analysis of all three peptide ions revealed that each peptide actually contained two species of modification by a single phosphorylation site. One species contained the modification site at Ser-102 (S1 site) and the other species contained the modification site at Ser-104 (S2 site). Here, we ascertained the assignments of both sides by careful analysis of the MS/MS spectra of the above three peptides. In the MS/MS spectrum of the modified peptide spanning 101–111 aa (A, top) two species of fragment ions (b or y ions) were shown to consider phosphorylation site either at Ser-102 (S1 site) or Ser-104 (S2 site). The spectra shows consecutive b ions due beta-elimination H3PO4 as b2-P (s1), b3-P (s1), b4-P (s1/s2) at 183.11 m/z, 298.13 m/z, 385.17, and 498.25 m/z, which indicated the phosphorylation site at Ser-102. The spectrum also showed relatively low intense a2+P (s1) peak at 253.09 m/z having the intact phosphate moiety. This provided significant confidence to assign the phosphorylation site at Ser-102. In addition, the intense y9-NH3 ion at 984.53 m/z corresponded to the unmodified peptide. This further confirmed the modification at Ser-102. On the other hand, the spectra showed b2 (s2) and b3 (s2) ions, respectively at 201.12 m/z and 316.15 m/z, which corresponded to unmodified peptide. This suggested some species of the peptide were not modified at Ser-102. However, the spectra showed b4-P, b5-P, b8-P and b10-P ions at 385.17 m/z, 498.25 m/z, 868.52 m/z and 1070.58 m/z, respectively due to possible beta-elimination of H3PO4 from Ser-104, suggesting the possible location of the phosphorylation site in other species of the peptide (101–111 aa) was at Ser-104. The MS/MS spectrum of tryptic missed cleaved peptide (101–112 aa) (B, top) showed similar fragmentation pattern as that of tryptic peptide spanning 101–111 aa (A, top). However, the a2+P (s1) ions at 253.09 m/z were more intense as compared to that of peptide spanning 101–111 aa (A, top), further suggesting the phosphorylation site at Ser-102. Finally, MS/MS spectrum of the other missed cleaved modified peptide (100–111 aa) (C, top), showed intense of b4 ion at 472.25 m/z corresponded to the unmodified peptide, suggesting no phosphorylation at Ser-102. However, the doubly charged y12 ion at 719.37 m/z contained the intact phosphate moiety. Thus the modification site in this peptide was assigned to Ser-104 as reported previously (Huq et al, 2005). Taken together, the analysis of the above three peptides revealed that both Ser-102 and Ser-140 are modified by mono-phosphorylation on RIP140.
(1.84 MB TIF)

Figure S3 PKCε distribution and functionality in differentiated adipocytes. In vitro phosphorylation of bacterial purified RIP140 by partially purified endogenous PKCε from nuclear and cytoplasmic fraction.
(0.30 MB TIF)
