# Supplementary figures and images for: PKCε Stimulated Arginine Methylation of RIP140 for Its Nuclear-Cytoplasmic Export in Adipocyte Differentiation
Source: PLoS One. 2008 Jul 16;3(7):e2658. doi: 10.1371/journal.pone.0002658 (PMC2440817; doi:10.1371/journal.pone.0002658)

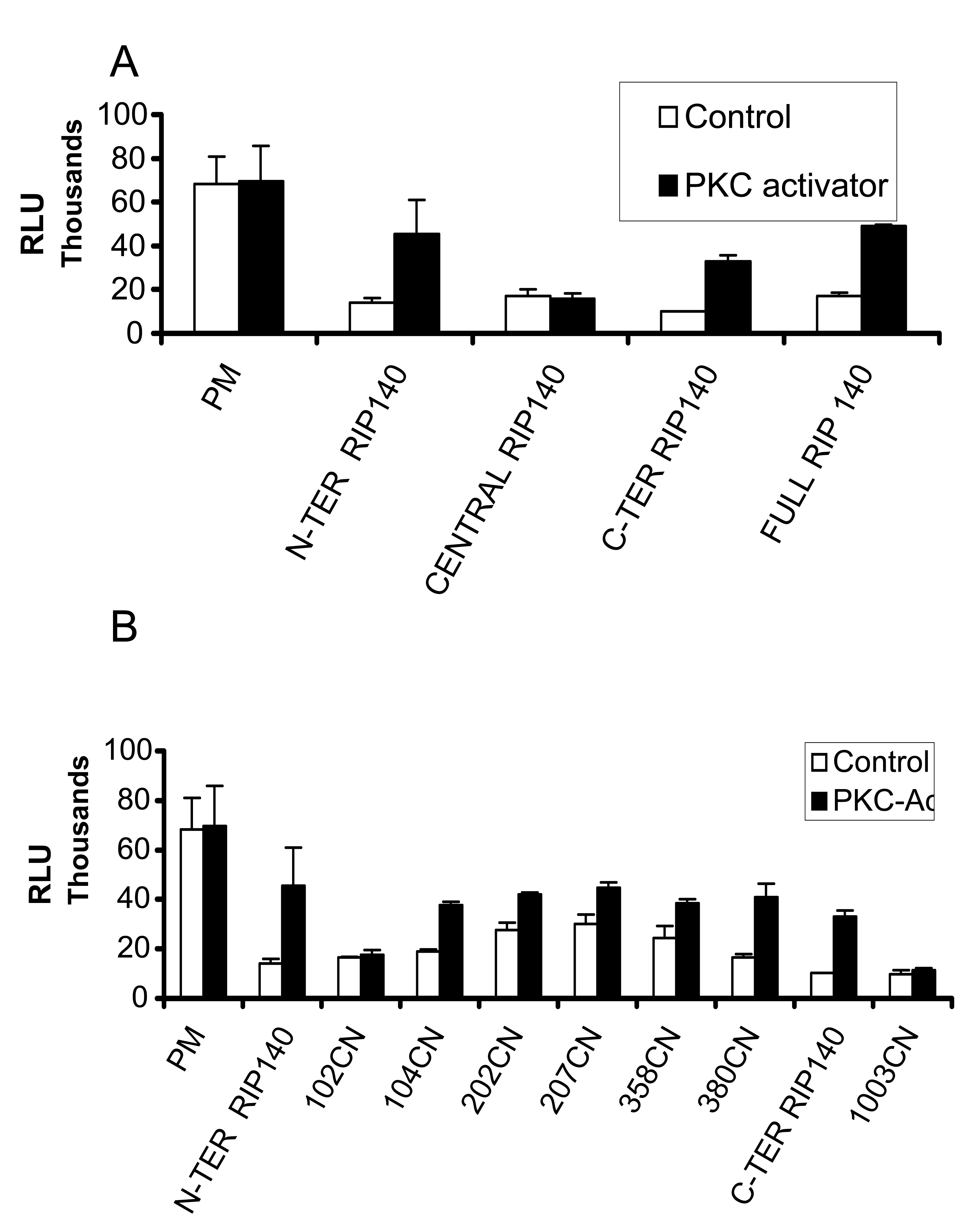

Supplement: Figure S1 — Screening of PKC responsive domains and residues of RIP140 with regards to its trans-repressive activity. (A) PKC activation reduced transrepression mediated by the N- and C-terminal, but not the central, domain of RIP140. (B) Ser-102 (in the N-terminal domain) and Ser-1003 (in the C-terminal domain) were responsive to PKC modulation. PKC activator: PMA. (0.23 MB TIF) [file pone.0002658.s001.tif]

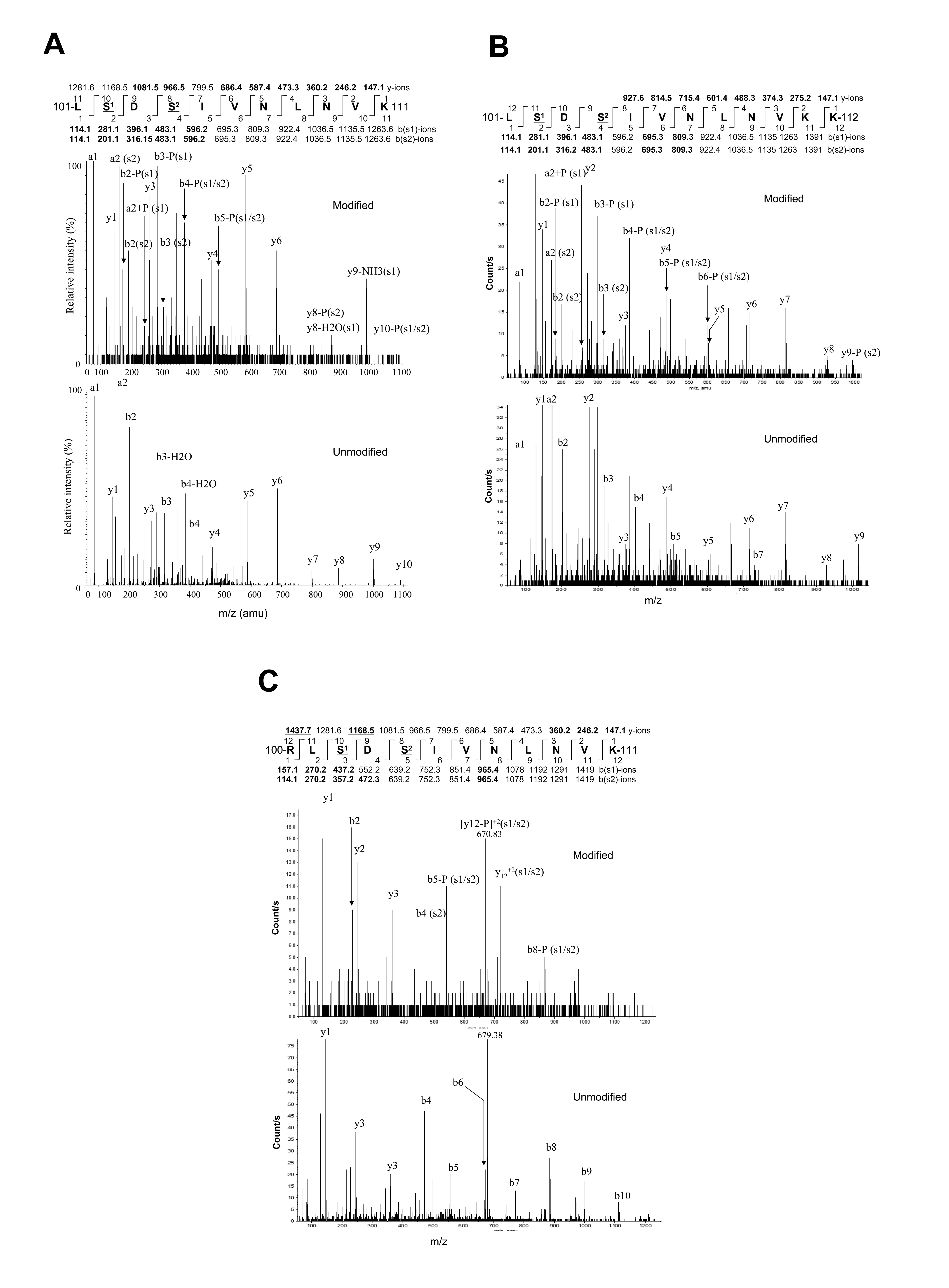

Supplement: Figure S2 — Mapping of phosphorylation sites on Ser-102 and Ser-104 on mouse RIP140 purified from insect cultures, by LC-ESI-MS/MS analysis. The total ion chromatogram (LC-MS) of tryptic digests of RIP140 showed three modified peptides spanning amino acids 101–111aa (101LSDSIVNLNVK111) (A), 101–112 aa (101LSDSIVNLNVKK112) (B), and 100–111 aa (100RLSDSIVNLNVK111) (C) contained both Ser-102 and Ser-104 (bold letter). The modified peptide spanning 100–111 (C, top) and 101–112 (B, top) appeared as doubly charged ions, respectively at 719.36 m/z (mol. mass 1436.72) and 705.35 m/z (mol. mass 140.72), while the peptide (101–111 aa) appeared as a triply charged ion at 641.31 m/z (mol. mass 1280.63). The precursor mass of each ion from the modified peptide showed +80 Da mass shift as compared to the each doubly charged peptide ion of the corresponding unmodified peptide 100–111 aa (679.37 m/z, mol. mass 1356.76 Da) (C, bottom), 101–111 aa (601.33 m/z, mol. mass 1200.66 Da) (A, bottom) and 101–112 aa (665.36 m/z, mol. mass 1328.75 Da) (B, bottom). This indicated that each peptide is modified by a mono-phosphorylation site. Previously, by MS/MS analysis of the precursor ion of the modified peptide 100–111 aa (C, top), we have reported the assignments of phosphorylation site at Ser-104 (Huq et al, 2005). However, careful analysis of all three peptide ions revealed that each peptide actually contained two species of modification by a single phosphorylation site. One species contained the modification site at Ser-102 (S1 site) and the other species contained the modification site at Ser-104 (S2 site). Here, we ascertained the assignments of both sides by careful analysis of the MS/MS spectra of the above three peptides. In the MS/MS spectrum of the modified peptide spanning 101–111 aa (A, top) two species of fragment ions (b or y ions) were shown to consider phosphorylation site either at Ser-102 (S1 site) or Ser-104 (S2 site). The spectra shows consecutive b ions due beta-elimination H3PO4 [file pone.0002658.s002.tif]

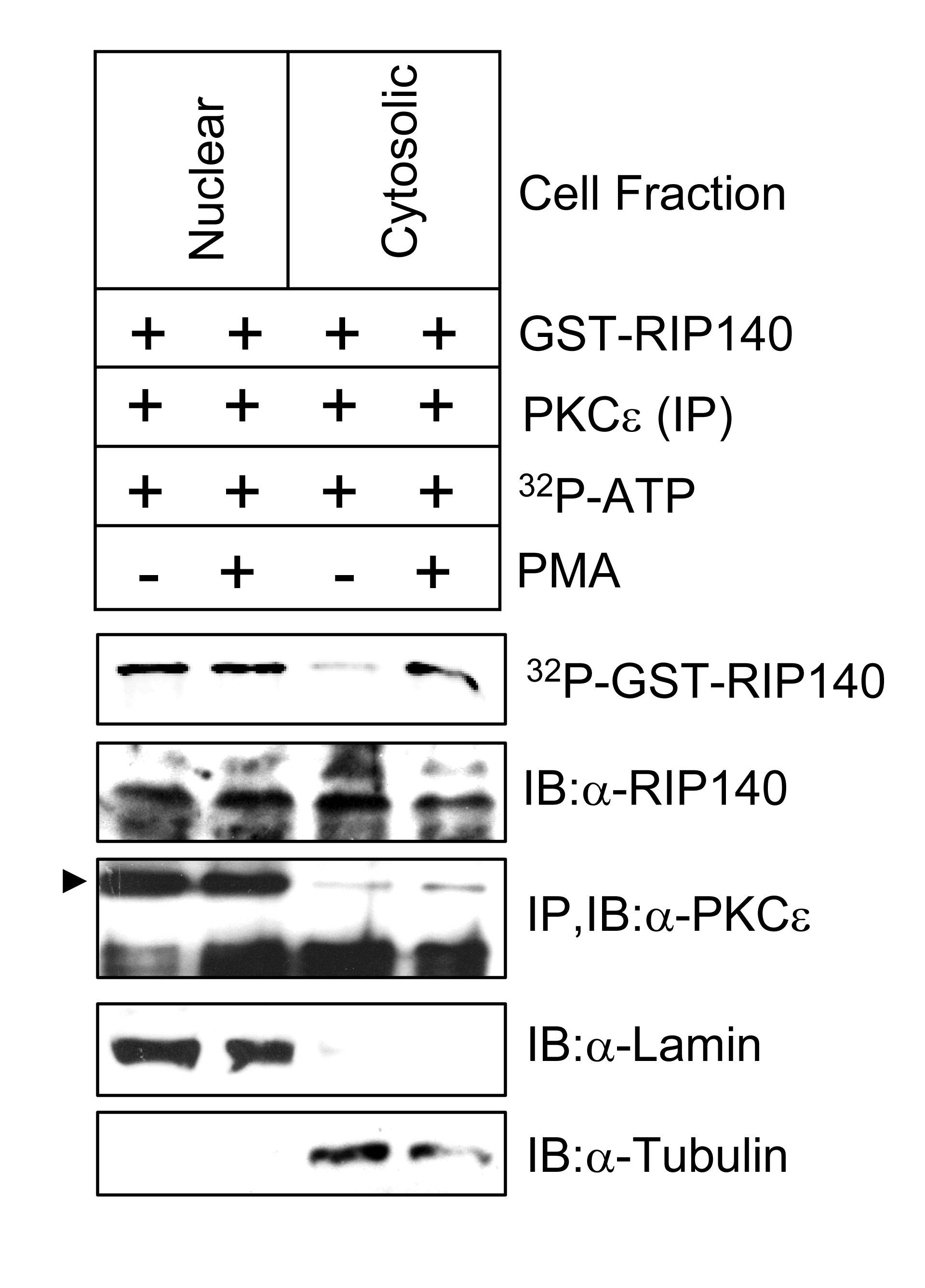

Supplement: Figure S3 — PKCε distribution and functionality in differentiated adipocytes. In vitro phosphorylation of bacterial purified RIP140 by partially purified endogenous PKCε from nuclear and cytoplasmic fraction. (0.30 MB TIF) [file pone.0002658.s003.tif]
